# Poster Session I - A37 ACTIVATED B CELL RESPONSES IN THE COLON OF ULCERATIVE COLITIS PATIENTS AND MOUSE MODEL OF COLITIS

**DOI:** 10.1093/jcag/gwaf042.037

**Published:** 2026-02-13

**Authors:** M Sabzevary, F Olayinka-Adefemi, A Marshall, J Ghia

**Affiliations:** Immunology, University of Manitoba Max Rady College of Medicine, Winnipeg, MB, Canada; Immunology, University of Manitoba Max Rady College of Medicine, Winnipeg, MB, Canada; Immunology, University of Manitoba Max Rady College of Medicine, Winnipeg, MB, Canada; Immunology, University of Manitoba Max Rady College of Medicine, Winnipeg, MB, Canada

## Abstract

**Background:**

IgA-producing B cells maintain gut homeostasis and control the entry of commensal bacteria and pathogens. Different B cell subtypes are expanded in the colon of patients with ulcerative colitis (UC). In a mouse model of colitis, activated markers have been detected on a large proportion of gut B cells. Activation signals driven by different receptors on B cells are primarily dependent on the phosphoinositide 3-kinase δ (PI3Kδ) pathway.

**Aims:**

To determine the effects of colonic inflammation on the activated B cell responses in UC patients and in a preclinical model of UC.

**Methods:**

Human transcriptomic data from UC patient and control colon biopsies were used for single-cell RNA sequencing analysis. Mice with a gain-of-function (GOF) mutation in PI3Kδ were crossed with Mb1-Cre transgenic mice to generate Cre^+^ mice with hyperactive B cells and Cre^-^ littermates as controls. Thirty six male Cre^+^ and Cre^-^ mice were treated with 1.5% (w/v) dextran sulfate sodium (DSS) for 6 days, followed by 8 days of recovery (regular water). Disease activity index was monitored daily. B cell phenotypes were assessed by flow cytometry in the Peyer’s patches and mesenteric lymph nodes (MLN). Serum levels of inflammatory and anti-inflammatory cytokines were measured by mesoscale. Levels of IgA, M, G1, and G2b in the colonic lumen and serum were determined by ELISA.

**Results:**

In UC patients, a large subset of mature B cells overexpressed CD86, which was coexpressed with HLA-DRB5, HLA-DPA1, and HLA-DQA1. Gene ontology analysis of mature B cells in UC patients also indicated enrichment in pathways related to mitochondrial ATP synthesis and B cell receptor signalling. In the preclinical model of colitis, PI3Kδ GOF Cre^+^ mice with hyperactive B cells exhibited faster recovery and gained weight compared to Cre^-^ littermates. Serum levels of IL-10 were significantly higher in colitic Cre^+^ compared to the colitic Cre^-^ groups, but there was no statistical difference in TNF-α, IFN-γ, and IL-12/IL-23p40 serum levels. Flow cytometry data showed a significant increase in the percentage of IL-10^+^ B cells in the MLNs and Peyer’s patches of Cre^+^ compared to the Cre^-^ mice. Furthermore, Cre^+^ mice in colitic condition had more IgM and IgG1&2b subclasses and less IgA levels in the colonic lumen and serum compared to the colitic Cre^-^ mice.

**Conclusions:**

Intestinal inflammation in UC patients leads to mature B cell activation with the antigen-presenting phenotype and mitochondrial respiration. In acute colitis, constitutive PI3Kδ activation in B cells accelerates recovery by inducing a subset of B cells to produce anti-inflammatory cytokines and antibodies, which may support intestinal immune protection. These findings may guide the development of new UC therapies.

**Funding Agencies:**

CAG, CCC

